# Hamiltonian energy and coexistence of hidden firing patterns from bidirectional coupling between two different neurons

**DOI:** 10.1007/s11571-021-09747-1

**Published:** 2021-12-02

**Authors:** Zeric Tabekoueng Njitacke, Bernard Nzoko Koumetio, Balamurali Ramakrishnan, Gervais Dolvis Leutcho, Theophile Fonzin Fozin, Nestor Tsafack, Kartikeyan Rajagopal, Jacques Kengne

**Affiliations:** 1 Department of Electrical and Electronic Engineering, College of Technology (COT), University of Buea, P.O. Box 63, Buea, Cameroon; 2 Research Unit of Automation and Applied Computer (URAIA), Electrical Engineering Department of IUT-FV, University of Dschang, P.O. Box 134, Bandjoun, Cameroon; 3 Department of Automation, Biomechanics and Mechatronics, Lodz University of Technology, Lodz, Poland; 4 Research Unit of Condensed Matter, Department of Physics, Faculty of Sciences, Electronics and Signal Processing (UR-MACETS), University of Dschang, P.O. Box 67, Dschang, Cameroon; 5 Center for Nonlinear Systems, Chennai Institute of Technology, Chennai, Tamil Nadu India; 6 Department of Electrical Engineering, École de Technologie Supérieure (ÉTS), Montréal, Québec H3C1K3 Canada; 7 Department of Electrical and Electronic Engineering, Faculty of Engineering and Technology (FET), University of Buea, P.O. Box 63, Buea, Cameroon

**Keywords:** Hindmarsh-Rose neuron, FitzHugh-Nagumo neuron, Asymmetric electrical synapse, Hamilton energy, Coexistence of hidden firing patterns, Pspice/Microcontroller implementation

## Abstract

In this paper, bidirectional-coupled neurons through an asymmetric electrical synapse are investigated. These coupled neurons involve 2D Hindmarsh–Rose (HR) and 2D FitzHugh–Nagumo (FN) neurons. The equilibria of the coupled neurons model are investigated, and their stabilities have revealed that, for some values of the electrical synaptic weight, the model under consideration can display either self-excited or hidden firing patterns. In addition, the hidden coexistence of chaotic bursting with periodic spiking, chaotic spiking with period spiking, chaotic bursting with a resting pattern, and the coexistence of chaotic spiking with a resting pattern are also found for some sets of electrical synaptic coupling. For all the investigated phenomena, the Hamiltonian energy of the model is computed. It enables the estimation of the amount of energy released during the transition between the various electrical activities. Pspice simulations are carried out based on the analog circuit of the coupled neurons to support our numerical results. Finally, an STM32F407ZE microcontroller development board is exploited for the digital implementation of the proposed coupled neurons model.

## Introduction

The human brain is an organ that can exhibit extremely complex nonlinear behavior (Natarajan et al. 2004). It is constituted of a huge number of interconnected neurons. Neurons can be viewed as the elementary, structural, and functional elements of the central nervous system (Tsou et al. 1998). Neurons play an important role in the processes of recording, selection, storage, and data transfer in the brain’s activities (Leuthardt et al. 2004). To ease the understanding of the dynamics of electrical activities in the brain, several mathematical models of neurons have been proposed and investigated in the literature, among which the Hodgkin–Huxley neuron (Hodgkin and Huxley 1952), Izhikevich neuron (Izhikevich 2003a), Morris–Lecar neuron (Tsumoto et al. 2006), 2-D and 3-D Hindmarsh–Rose (HR) model (Hindmarsh and Rose 1982, 1984), FitzHugh–Nagumo (FN) model (Izhikevich and FitzHugh 2006), Hopfield neural network model (Njitacke et al. 2020a, 2020b; Doubla Isaac et al. 2020; Tabekoueng Njitacke et al. 2020) and Chay model (Chay 1985) just to name few. These various neurons have been intensively studied separately as well as coupled. Concerning the investigation of the single neuron, (Zhang et al. 2021) introduced a novel free-equilibrium HR model using memristive electromagnetic induction. During their investigations, the authors found that the proposed model of the neuron is capable of unusual and rare phenomena of hidden homogeneous extreme multistability, defined by the coexistence of an infinite number of firing activities with the same shape but at different positions. It is good to mention that such types of nonlinear behaviors involving multistability, hidden dynamics previously found in neurons were also able to occur in circuits and systems, such as the extended Lu system(Lai et al. 2018a), a unified chaotic system (Lai 2021), a two-memristor-based chaotic system (Lai et al. 2020a) as well as a non-equilibrium chaotic system (Lai et al. 2020b). In (Ngouonkadi et al. 2016), the authors studied in an extended model of a 4D HR neuron model the phenomenon of coexistence of firing patterns. The investigations revealed that the model is able to exhibit several types of complex phenomena, including symmetry breaking, period doubling, reverse period-doubling, and crisis scenarios. Hou et al. (Hou et al. 2021) have estimated the electrical activity in a neuron under a depolarization field. During their investigation, they found that there was a peak in the firing interval of neurons with the increase of stimulation current intensity. In the presence of an electric field, the firing pattern of the neuron was transformed from single bursting to intermittent multimodal bursting with the increase in frequency and amplitude of the electric field. Kafraj et al. (Kafraj et al. 2020) have introduced and investigated a three-variable memristive Izhikevich model to describe the behavior of neurons under electromagnetic induction and noise. Their model represented the effect of internal and external magnetic fields on neurons. The improved model without the external magnetic field was able to exhibit firing patterns such as regular spiking, resonator, chattering, fast-spiking, chaotic spiking, and chaotic bursts. The presence of the external magnetic field was able to change the firing pattern of the neuron, for example, shifting from chaotic firings to periodic ones or vice versa. It was also observed that the external field effect stimulated the neuron to fire, while in the absence of the external field, it was at rest.

Besides the above-mentioned advances, investigation of the coupled neurons has also attracted a lot of attention. For example, Pisarchik et al. (Pisarchik et al. 2018) explored the dynamics of two identical 3D HR coupled models through an asymmetric electrical coupling. The result indicated that the 6D neuronal oscillator obtained was able to exhibit the phenomenon of multistability with up to six firing activities. In a real brain, it is very difficult to have a connection between two identical neurons with identical parameters. Henceforth, Njitacke et al. (Tabekoueng Njitacke et al. 2020) have considered the dynamics of two coupled 2D HR neurons with a small discrepancy of their parameters. From their investigations, the authors found that their proposed simple 4D neural oscillator was able to exhibit traditional brain firing activities including bursting, spiking oscillations as well as the phenomenon of bistability under the variation of the symmetric coupling weight and the external stimulus. In (Wang et al. 2016) the authors coupled the neurons through various chemical synapses, namely: rapid excitatory and inhibitory synapses. The spatiotemporal patterns were analyzed in the coupled model. The results indicated that the rapid excitatory synapse coupling type is easier to produce periodic spatiotemporal patterns than the rapid inhibitory synapse coupling type, and the process was analyzed using the bifurcation of a unique neuron model. They also introduced the permutation entropy, defined as a measure of network firing complexity to explore the process of formation and transition of spatiotemporal patterns. Karthikeyan et al. (Rajagopal et al. 2020) have introduced a modified Hindmarsh-Rose neuron model having a fractional-order threshold magnetic flux. Their investigations were conducted both in the presence and absence of external electromagnetic induction. Besides, the emergence of the spiral waves in the network of the proposed model was studied. To find the effects of different factors on the formation and destruction of spiral waves, the external current, the coupling strength, and the external stimulus amplitude were varied. It has been observed that all of these parameters have significant impacts on the spiral waves.

As we all know, the nervous system has a large number of neurons with different biological structures and functions. Most memristive neurons and neural network models consider only identical neurons and a single biological function (Hou et al. 2021; Tabekoueng Njitacke et al. 2020; Goetze and Lai 2021; Joshi 2021; Mersing et al. 2021; Wouapi et al. 2021). Then, the previous literature is devoted to investigating the electrical activities of coupled neurons with either identical neurons or non-identical neurons governed by the same mathematical model. However, the dynamical behaviors of coupled neurons with different mathematical models were rarely reported in previous publications (Li et al. 2021). Thus, more different memristive neuron and neural network models need to be developed based on the different biological neuronal systems as well as different mathematical models (Lin et al. 2021).

Very recently, Li et al. (Li et al. 2021) reported the dynamics of coupled neurons made of HR and FN models under the magnetic field effect. Studies of the coupled neurons showed the phenomenon of firing pattern coexistence as well as phase synchronization. Before performing this work on coupled neurons consisting of HR and FN models under the effect of the magnetic field, it would have been interesting to see what happened to the original 2D HR neuron coupled to the original 2D FN neuron by asymmetric electrical synapses. This later questioning justifies the interest of this work since in a normal brain it is difficult to have a homogenous coupling between neurons as presented in Refs. (Tabekoueng Njitacke et al. 2020; Bao et al. 2019a; Ren et al. 2017; Njitacke et al. 2020).

It is well known that during the metabolic process of the neuronal system or biological system, energy is consumed so that neurons can save normal and continuous electric activity (Harris et al. 2012). The liberation and storage of energy of neuronal models can be estimated. Then it is interesting to detect the energy transmission and release dependencies on the electric activity mode in those neuronal models. In several works focused on the dynamics of neurons, Hamilton’s statistical function is usually exploited to the energy consumption by exploiting Helmholtz’s theorem. The said energy can be also exploited to explain some biological phenomena such as synchronization regime, spiking state, bursting state. This discussion on the energy of the neurons enables us to see the main drawback of the previous works addressed on coupled neurons, and it is solved in this work.

Then, in the present contribution, we have investigated the complex dynamics of a simple heterogeneous neural network made of the coupling between a 2D HR neuron model with a 2D FN neuron model through asymmetric electrical synapses. In addition, the electrical activity and the Hamilton energy in the coupled neurons are analyzed by considering the simultaneous effect of the asymmetrical electrical coupling. Based on Helmholtz’s theorem, the Hamilton energy function *H* and its derivative in terms of membrane potentials, recovery variables, and parameters of the model are provided (Lu et al. 2019; Ma et al. 2017; Xin-Lin et al. 2015). This work is very distinct from the previous research works, which focus on the homogeneous coupling between neurons.

The plan of this work is presented as follows: In Sect. 2, the heterogeneous model of the coupled neurons is established. In Sect. 3, the dynamics of the coupled neurons model is studied and its Hamilton energy analysed. In Sect. 4, Pspice implementation of the coupled neurons is realized. In Sect. 5, the microcontroller implementation of the coupled neurons is addressed using the STM32F407ZE microcontroller development board. Finally, we summarize the paper in Sect. 6.

## The model and its basic dynamics studies

### Model description

**Fig. 1:**
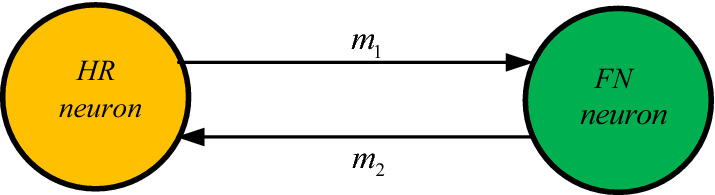
Bidirectional coupling between the two different families of 2D neurons

It is well known that biological neurons can develop several types of firing activities to reproduce brain dynamics. To achieve that goal several mathematical models of functional neurons have been proposed in the literature. Among others, we have the Hodgkin–Huxley neuron (Hodgkin and Huxley 1952), Hindmarsh–Rose (HR) neurons (Hindmarsh and Rose 1982), Morris–Lecar neuron (Tsumoto et al. 2006), Fitzhugh-Nagumo neuron (Izhikevich and FitzHugh 2006), Izhikevich neuron (Izhikevich 2003b), Hopfield neural networks (Njitacke et al. 2020a, 2019; Njitacke and Kengne 2018, 2019), and so on. Let us recall that reduced from the Hodgkin-Huxley neuron model (Hodgkin and Huxley 1952), the 2D HR neuron model was first proposed in 1982 (Hindmarsh and Rose 1982) and, is described as:

1 $$\left\{ \begin{gathered} \dot{x}_{1} = y_{1} - a_{1} x_{1}^{3} + b_{1} x_{1}^{2} + I_{1} \hfill \\ \dot{y}_{1} = c_{1} - d_{1} x_{1}^{2} - y_{1} \hfill \\ \end{gathered} \right.$$

where $x_{1}$, $y_{1}$ and $I_{1}$ respectively indicate the membrane potential, the spiking variable, and steady current. Besides another 2-D simplification of the Hodgkin-Huxley (HH) model leads to the FitzHugh-Nagumo (FN) model described by Izhikevich and FitzHugh (2006)

2 $$\left\{ \begin{gathered} \dot{x}_{2} = x_{2} - b_{2} x_{2}^{3} - y_{2} + I_{2} \hfill \\ \dot{y}_{2} = \frac{1}{\varepsilon }\left( {a_{2} + x_{2} - c_{2} y_{2} } \right) \hfill \\ \end{gathered} \right.$$

where $x_{2}$, $y_{2}$ and $I_{2}$ respectively indicate the membrane potential, the retrieval variable, and the magnitude of stimulus current. In the brain as well as in the neural system, coupling plays an essential role in the processes of generation of particular rhythms and formation of memory. However, in all the previous works, the authors have considered the coupling between identical neurons. Although in practice, the coupling of neurons can be done between different families of neurons having different mathematical models. Motivated by this observation, the bidirectional coupling between the HR neuron and the FN neuron through electrical synapses of Fig. 1 is considered in this work.

Based on that figure, the mathematical model of the coupled neurons is obtained as:

3 $$\left\{ \begin{gathered} \dot{x}_{1} = y_{1} - a_{1} x_{1}^{3} + b_{1} x_{1}^{2} + I_{1} + m_{1} \left( {x_{2} - x_{1} } \right) \hfill \\ \dot{y}_{1} = c_{1} - d_{1} x_{1}^{2} - y_{1} \hfill \\ \dot{x}_{2} = x_{2} - b_{2} x_{2}^{3} - y_{2} + I_{2} + m_{2} \left( {x_{1} - x_{2} } \right) \hfill \\ \dot{y}_{2} = {\raise0.7ex\hbox{$1$} \!\mathord{\left/ {\vphantom {1 \varepsilon }}\right.\kern-\nulldelimiterspace} \!\lower0.7ex\hbox{$\varepsilon $}}\left( {a_{2} + x_{2} - c_{2} y_{2} } \right) \hfill \\ \end{gathered} \right.$$

Here $$x_{1}$$ and $$x_{2}$$ are the potentials of the membrane in HR neuron and FN neuron respectively, $$y_{1}$$ and $$y_{2}$$ are retrieval variables related to a fast current of either $$Na^{ + }$$ or $$K^{ + }$$, $$i_{1}$$ and $$i_{2}$$ represent exterior input currents and $$m_{1}$$ and $$m_{2}$$ indicate the strengths of electrical coupling utilized as command parameters. Let us stress that in the considerations of this model the parameters are all positive and defined as: $$a_{1} = 1$$, $$b_{1} = 3.05$$, $$c_{1} = 1$$, $$d_{1} = 5$$,$$a_{2} = 0.77$$, $$b_{2} = {\raise0.7ex\hbox{$1$} \!\mathord{\left/ {\vphantom {1 3}}\right.\kern-\nulldelimiterspace} \!\lower0.7ex\hbox{$3$}}$$, $$c_{2} = 0.8$$$$\varepsilon = 13$$_,_ $$i_{1} = 0.4$$, $$m_{1} = 1$$, $$i_{2} = 0$$ and $$m_{2} = tuneable$$.

### Steady states and their stabilities

The steady states of our coupled neurons are obtained by solving the equation $\dot{x}_{1} = \dot{y}_{1} = \dot{x}_{2} = \dot{y}_{2} = 0$ thus we obtain

4 $$\left\{ \begin{gathered} y_{1} - a_{1} x_{1}^{3} + b_{1} x_{1}^{2} + I_{1} + m_{1} \left( {x_{2} - x_{1} } \right) = 0 \hfill \\ c_{1} - d_{1} x_{1}^{2} - y_{1} = 0 \hfill \\ - y_{2} + x_{2} - b_{2} x_{2}^{3} + I_{2} + m_{2} \left( {x_{1} - x_{2} } \right) = 0 \hfill \\ {\raise0.7ex\hbox{$1$} \!\mathord{\left/ {\vphantom {1 \varepsilon }}\right.\kern-\nulldelimiterspace} \!\lower0.7ex\hbox{$\varepsilon $}}\left( {a_{2} + x_{2} - c_{2} y_{2} } \right) = 0 \hfill \\ \end{gathered} \right.$$

For the considered coupled neurons, the steady states are numerically determined as

5 $$\left( {x_{1e} ,y_{1e} ,x_{2e} ,y_{2e} } \right) = \left( {\overline{x}_{1} ,c_{1} - d_{1} \overline{x}_{1}^{2} ,\overline{x}_{2} ,{\raise0.7ex\hbox{$1$} \!\mathord{\left/ {\vphantom {1 {c_{2} }}}\right.\kern-\nulldelimiterspace} \!\lower0.7ex\hbox{${c_{2} }$}}\left( {a_{2} + \overline{x}_{2} } \right)} \right)$$

where $\overline{x}_{1}$ and $\overline{x}_{2}$ can be obtained graphically from the intersection between the following curves

6 $$f_{1} \left( {\overline{x}_{1} ,\overline{x}_{2} } \right) = \overline{x}_{1} + \frac{1}{{m_{2} }}\left( { - \left( {{\raise0.7ex\hbox{$1$} \!\mathord{\left/ {\vphantom {1 {c_{2} }}}\right.\kern-\nulldelimiterspace} \!\lower0.7ex\hbox{${c_{2} }$}}} \right)\left( {a_{2} + x_{2} } \right) - x_{2} - b_{2} x_{2}^{3} + I_{2} - m_{2} x_{2} } \right) = 0$$

7 $$f_{2} \left( {\overline{x}_{1} ,\overline{x}_{2} } \right) = \overline{x}_{2} + \frac{1}{{m_{1} }}\left( {c_{1} - d_{1} \overline{x}_{1}^{2} - a_{1} \overline{x}_{1}^{3} + b_{1} \overline{x}_{1}^{2} + I_{1} - m_{1} \overline{x}_{1} } \right) = 0$$

**Fig. 2:**
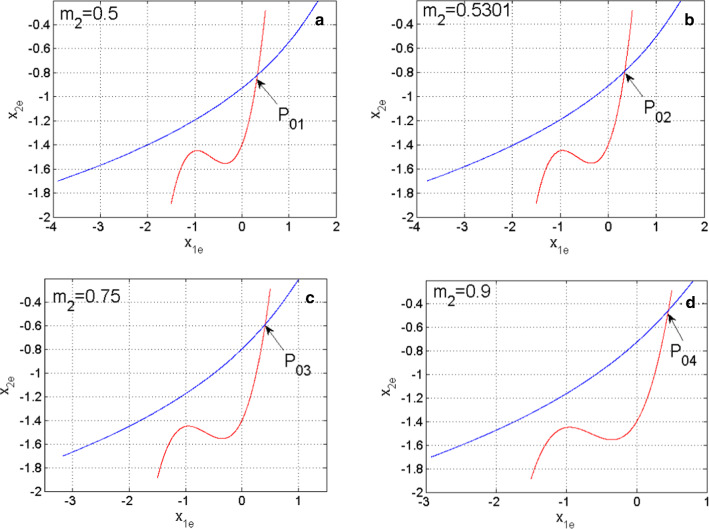
Evolution of the functions defined In Eq. 6 and Eq. 7 showing the steady states of the coupled neurons at the intersection for some selected values of synaptic weight $m_{2}$

Considering some selected values of the synaptic weight $m_{2}$ the steady states of the coupled neurons model are shaped in Fig. 2. From this figure, it is evident that the coupled neurons model possesses only one steady state since both curves display one intersection point. It is also observed that the stability of the steady-state depends on the control synaptic weight $m_{2}$. To have an idea of the stability of the coupled neurons, let consider the following Jacobian matrix:

8 $$J = \left[ {\begin{array}{*{20}c} { - 3a_{1} \overline{x}_{1}^{2} + 2b_{1} \overline{x}_{1} - m_{1} } & 1 & {m_{1} } & 0 \\ { - 2d_{1} \overline{x}_{1} } & { - 1} & 0 & 0 \\ {m_{2} } & 0 & { - 3b_{2} \overline{x}_{2}^{2} + 1 - m_{2} } & { - 1} \\ 0 & 0 & {{\raise0.7ex\hbox{$1$} \!\mathord{\left/ {\vphantom {1 \varepsilon }}\right.\kern-\nulldelimiterspace} \!\lower0.7ex\hbox{$\varepsilon $}}} & {{\raise0.7ex\hbox{${ - c_{2} }$} \!\mathord{\left/ {\vphantom {{ - c_{2} } \varepsilon }}\right.\kern-\nulldelimiterspace} \!\lower0.7ex\hbox{$\varepsilon $}}} \\ \end{array} } \right]$$

The eigenvalues at the steady-state $\left( {\overline{x}_{1} ,\overline{y}_{1} ,\overline{x}_{2} ,\overline{y}_{2} } \right)$ are computed from the following equation:

9 $$\det \left( {\lambda I - J} \right) = 0$$

Using some selected values of the electrical synaptic weight $m_{2}$, the steady states and their stability are summarized in Table 1. From Table 1 it is evident that the coupled neurons can display either hidden dynamics associated with a stable equilibrium point or self-excited dynamics associated with unstable equilibria (Njitacke et al. 2020a; Lai et al. 2018b; Tsafack et al. 2020).

**Table 1:** Steady states for some values of $m_{2}$ with their corresponding eigenvalues and stabilities

Electrical synapse value	Steady states	Eigenvalues	System stability
$m_{2} = 0.5$	$P_{01} = \left( {{ 0}{\text{.3311,0}}{.4519, - 0}{\text{.8186, - 0}}{.0607}} \right)$	$$\begin{gathered} { - 5}{\text{.6884 + 0}}{\text{.0000i}} \hfill \\ { - 0}{\text{.1190 }} \pm {1}{\text{.5769i}} \hfill \\ { - 0}{\text{.0758 + 0}}{\text{.0000i}} \hfill \\ \end{gathered}$$	Stable
$m_{2} = 0.5301$	$P_{02} = \left( {{0}{\text{.3417,0}}{.4162, - 0}{\text{.7904, - 0}}{.0255}} \right)$	$$\begin{gathered} { - 5}{\text{.3093 + 0}}{\text{.0000i}} \hfill \\ { - 0}{\text{.0938}} \pm {1}{\text{.5925i}} \hfill \\ { - 0}{\text{.0770 + 0}}{\text{.0000i}} \hfill \\ \end{gathered}$$	Stable
$m_{2} = 0.75$	$P_{03} = \left( {{0}{\text{.4123,0}}{.1500, - 0}{\text{.5861,0}}{.2299}} \right)$	$$\begin{gathered} { - 2}{\text{.9905 + 0}}{\text{.0000i}} \hfill \\ {0}{\text{.0661 }} \pm {1}{\text{.6547i}} \hfill \\ { - 0}{\text{.0913 + 0}}{\text{.0000i}} \hfill \\ \end{gathered}$$	Unstable
$m_{2} = 0.9$	$P_{04} = \left( {{0}{\text{.4525, - 0}}{.0238, - 0}{\text{.4554,0}}{.3932}} \right)$	$$\begin{gathered} { 0}{\text{.1328}} \pm {1}{\text{.6399i}} \hfill \\ { - 1}{\text{.8641 + 0}}{\text{.0000i}} \hfill \\ { - 0}{\text{.1146 + 0}}{\text{.0000i}} \hfill \\ \end{gathered}$$	Unstable
$m_{2} = 1.0$	$P_{05} = \left( {{0}{\text{.4757, - 0}}{.1315, - 0}{\text{.3753,0}}{.4934}} \right)$	$$\begin{gathered} {0}{\text{.1477}} \pm {1}{\text{.6126i}} \hfill \\ { - 0}{\text{.1466 + 0}}{\text{.0000i}} \hfill \\ { - 1}{\text{.2761 + 0}}{\text{.0000i}} \hfill \\ \end{gathered}$$	Unstable
$m_{2} = 0.54$	$P_{06} = \left( {{0}{\text{.3452,0}}{.4042, - 0}{\text{.7812, - 0}}{.0140}} \right)$	$$\begin{gathered} { - 5}{\text{.1889 + 0}}{\text{.0000i}} \hfill \\ { - 0}{\text{.0855}} \pm {1}{\text{.5975i}} \hfill \\ { - 0}{\text{.0774 + 0}}{\text{.0000i}} \hfill \\ \end{gathered}$$	Stable
$m_{2} = 0.523$	$P_{07} = \left( {{0}{\text{.3393,0}}{.4244, - 0}{\text{.7971, - 0}}{.0339}} \right)$	$$\begin{gathered} { - 5}{\text{.3981 + 0}}{\text{.0000i}} \hfill \\ { - 0}{\text{.0995 }} \pm {1}{\text{.5891i}} \hfill \\ { - 0}{\text{.0767 + 0}}{\text{.0000i}} \hfill \\ \end{gathered}$$	Stable

### Dissipation property

The model of the coupled neurons is dissipative if its divergence $\nabla \phi$ is negative i.e. $\nabla \phi < 0$. The divergence of the coupled neurons model defined in Eq. (3) also known as the volume contraction rate is evaluated as indicated in Eq. (10) to evaluate the dissipation property.

10 $$\nabla \phi = \frac{{\partial \dot{x}_{1} }}{{\partial x_{1} }} + \frac{{\partial \dot{y}_{1} }}{{\partial y_{1} }} + \frac{{\partial \dot{x}_{2} }}{{\partial x_{2} }} + \frac{{\partial \dot{y}_{2} }}{{\partial y_{2} }} = - \left( {3a_{1} x_{1}^{2} + 3b_{2} x_{2}^{2} + m_{1} + m_{2} + \frac{{c_{2} }}{\varepsilon }} \right) + 2b_{1} x_{1}$$

where $\phi$ stands for the phase volume. To be sure that coupled model of neurons is dissipative, the result of the divergence should verify $- \left( {3a_{1} x_{1}^{2} + 3b_{2} x_{2}^{2} + m_{1} + m_{2} + \frac{{c_{2} }}{\varepsilon }} \right) + 2b_{1} x_{1} < 0$ which imply that $2b_{1} x_{1} < \left( {3a_{1} x_{1}^{2} + 3b_{2} x_{2}^{2} + m_{1} + m_{2} + \frac{{c_{2} }}{\varepsilon }} \right)$. This enables the volume element $\phi_{0}$ to be contracted and the flow to be confined into a volume element $\phi_{0} e^{{ - \left( {\left( {3a_{1} x_{1}^{2} + 3b_{2} x_{2}^{2} + m_{1} + m_{2} + \frac{{c_{2} }}{\varepsilon }} \right) - 2b_{1} x_{1} } \right)t}}$ over time $t$. In other words when $t \to \infty$, each volume involving the trajectories of the coupled neurons model contract to zero with the exponential rate $- \left( {\left( {3a_{1} x_{1}^{2} + 3b_{2} x_{2}^{2} + m_{1} + m_{2} + \frac{{c_{2} }}{\varepsilon }} \right) - 2b_{1} x_{1} } \right)$. Consequently, the trajectories are all confined in a particular set of zero volume and the behavior is stabilized on a stable state in the state space (Zheng et al. 2004).

### Hamiltonian energy of the coupled neurons

The field of study based on energy utilization in the brain has been considered experimentally and not theoretically due to the complex connections in the brain (Chuankui 2011). Given that there is no accurate method to establish the energy consumption and supply, Hamilton energy is usually exploited to identify the appurtenance of state on energy considering a unique neuron model or a coupled neurons model (Nabi et al. 2012). To Determine the energy of our coupled neurons $F\left( x \right)$, let us express its dynamical equations by Eq. (11) (Lu et al. 2019; Xin-Lin et al. 2015)

11 $$F\left( x \right) = F_{c} \left( x \right) + F_{d} \left( x \right) = \left[ {J\left( x \right) + R\left( x \right)} \right]\nabla H$$

here $F_{c} \left( x \right)$ and $F_{d} \left( x \right)$ represent the conservative component and the dissipative component, respectively. $\nabla H$ represents the gradient matrix of an energy function $H\left( x \right)$. $J\left( x \right)$ Indicates a skew-symmetric matrix and $R\left( x \right)$ is a symmetric matrix. The Hamilton energy function can be determined as:

12 $$\begin{gathered} \left\{ \begin{gathered} \dot{H} = \nabla H^{T} R\left( x \right)\nabla H = \nabla H^{T} F_{d} \left( x \right) \hfill \\ \nabla H^{T} J\left( x \right)\nabla H = \nabla H^{T} F_{c} \left( x \right) = 0 \hfill \\ \end{gathered} \right. \hfill \\ \hfill \\ \end{gathered}$$

Thus, for the coupled bidirectional neurons given in Eq. (3), we have

13 $$F_{c} = \left[ \begin{gathered} y_{1} + I_{1} + m_{1} x_{2} \hfill \\ c_{1} - d_{1} x_{1}^{2} \hfill \\ - y_{2} + I_{2} + m_{2} x_{1} \hfill \\ \frac{1}{\varepsilon }\left( {a_{2} + x_{2} } \right) \hfill \\ \end{gathered} \right]$$

14 $$F_{d} = \left[ \begin{gathered} - a_{1} x_{1}^{3} + b_{1} x_{1}^{2} - m_{1} x_{1} \hfill \\ - y_{1} \hfill \\ x_{2} - b_{2} x_{2}^{3} - m_{2} x_{2} \hfill \\ - \frac{{c_{2} }}{\varepsilon }y_{2} \hfill \\ \end{gathered} \right]$$

From Eqs. (11) and (12), the Hamilton energy function $H\left( {x_{1} ,y_{1} ,x_{2} ,y_{2} } \right)$ of the coupled neurons can be expressed as follows:

15 $$\left( {y_{1} + I_{1} + m_{1} x_{2} } \right)\frac{\partial H}{{\partial x_{1} }} + \left( {c_{1} - d_{1} x_{1}^{2} } \right)_{1} \frac{\partial H}{{\partial y_{1} }} + \left( { - y_{2} + I_{2} + m_{2} x_{1} } \right)\frac{\partial H}{{\partial x_{2} }} + \frac{1}{\varepsilon }\left( {a_{2} + x_{2} } \right)\frac{\partial H}{{\partial y_{2} }} = 0$$

The general solution of Eq. (15) is computed as:

16 $$\begin{aligned} H = & \frac{{ - 2d_{1} }}{{3m_{1} }}x_{1}^{3} \\ + & \frac{{2c_{1} }}{{m_{1} }}x_{1} + \frac{1}{{\varepsilon m_{2} }}\left( {a_{2} + x_{2} } \right)^{2} - \frac{1}{{m_{1} }}\left( {y_{1} + I_{1} + m_{1} x_{2} } \right)^{2} + \frac{1}{{m_{2} }}\left( { - y_{2} + I_{2} + m_{2} x_{1} } \right)^{2} \\ \end{aligned}$$

The derivative of the Hamilton energy function with respect to the time is given by

17 $$\begin{aligned} \dot{H} = & \dot{x}_{1} \frac{{ - 2d_{1} }}{{m_{1} }}x_{1}^{2} + \frac{{2c_{1} }}{{m_{1} }} + 2\left( { - y_{2} + I_{2} + m_{2} x_{1} } \right) + \dot{y}_{1} \left( { - \frac{2}{{m_{1} }}(y_{1} + I_{1} + m_{1} x_{2} )} \right) + \\ \dot{x}_{2} \left( { - 2(y_{1} + I_{1} + m_{1} x_{2} ) + \frac{2}{{\varepsilon m_{2} }}(a_{2} + x_{2} )} \right) + \dot{y}_{2} \left( {\frac{2}{{m_{2} }}( - y_{2} + I_{2} + m_{2} x_{s} )} \right) \\ \end{aligned}$$

18 $$\begin{gathered} \dot{H} = \left( { - a_{1} x_{1}^{3} + b_{1} x_{1}^{2} - m_{1} x_{1} + y_{1} + I_{1} + m_{1} x_{2} } \right)\left( {\frac{{ - 2d_{1} }}{{m_{1} }}x_{1}^{2} + \frac{{2c_{1} }}{{m_{1} }} + 2\left( { - y_{2} + I_{2} + m_{2} x_{1} } \right)} \right) + \left( { - y_{1} + c_{1} - d_{1} x_{1}^{2} } \right)\left( { - \frac{2}{{m_{1} }}\left( {y_{1} + I_{1} + m_{1} x_{2} } \right)} \right) + \hfill \\ \left( {x_{2} - b_{2} x_{2}^{3} - m_{2} x_{2} + m_{2} x_{1} + I_{2} - y_{2} } \right)\left( { - 2\left( {y_{1} + I_{1} + m_{1} x_{2} } \right) + \frac{2}{{\varepsilon m_{2} }}\left( {a_{2} + x_{2} } \right)} \right) + \left( {\frac{{ - c_{2} }}{\varepsilon }y_{2} + \frac{1}{\varepsilon }\left( {a_{2} + x_{2} } \right)} \right)\left( {\frac{ - 2}{{m_{2} }}\left( { - y_{2} + I_{2} + m_{2} x_{1} } \right)} \right) \hfill \\ \end{gathered}$$

After some algebraic manipulation, we obtained

19 $$\begin{gathered} \dot{H} = \left( {\frac{{ - 2d_{1} }}{{m_{1} }}x_{1}^{2} + \frac{{2c_{1} }}{{m_{1} }} + 2\left( { - y_{2} + I_{2} + m_{2} x_{1} } \right)} \right)\left( { - a_{1} x_{1}^{3} + b_{1} x_{1}^{2} - m_{1} x_{1} } \right) + \left( { - \frac{2}{{m_{1} }}\left( {y_{1} + I_{1} + m_{1} x_{2} } \right)} \right)\left( { - y_{1} } \right) + \hfill \\ \, \left( { - 2\left( {y_{1} + I_{1} + m_{1} x_{2} } \right) + \frac{2}{{\varepsilon m_{2} }}\left( {a_{2} + x_{2} } \right)} \right)\left( {x_{2} - b_{2} x_{2}^{3} - m_{2} x_{2} } \right) + \left( {\frac{ - 2}{{m_{2} }}\left( { - y_{2} + I_{2} + m_{2} x_{1} } \right)} \right)\left( { - \frac{{c_{2} }}{\varepsilon }y_{2} } \right) \hfill \\ \end{gathered}$$

In addition,

20 $$\nabla H^{T} F_{d} = \left[ {\begin{array}{*{20}c} {\gamma_{1} } & {\gamma_{2} } & {\gamma_{3} } & {\gamma_{4} } \\ \end{array} } \right]\left[ \begin{gathered} - a_{1} x_{1}^{3} + b_{1} x_{1}^{2} - m_{1} x_{1} \hfill \\ - y_{1} \hfill \\ x_{2} - b_{2} x_{2}^{3} - m_{2} x_{2} \hfill \\ - \frac{{c_{2} }}{\varepsilon }y_{2} \hfill \\ \end{gathered} \right]$$

21 $$\begin{gathered} \gamma_{1} = \frac{{ - 2d_{1} }}{{m_{1} }}x_{1}^{2} + \frac{{2c_{1} }}{{m_{1} }} + 2\left( { - y_{2} + I_{2} + m_{2} x_{1} } \right), \, \gamma_{2} = - \frac{2}{{m_{1} }}\left( {y_{1} + I_{1} + m_{1} x_{2} } \right) \hfill \\ \gamma_{3} = - 2\left( {y_{1} + I_{1} + m_{1} x_{2} } \right) + \frac{2}{{\varepsilon m_{2} }}\left( {a_{2} + x_{2} } \right){, }\gamma_{4} { = }\left. {\frac{ - 2}{{m_{2} }}\left( { - y_{2} + I_{2} + m_{2} x_{1} } \right)} \right) \, \hfill \\ \end{gathered}$$

Then

22 $$\begin{gathered} \nabla H^{T} F_{d} = \left( {\frac{{ - 2d_{1} }}{{m_{1} }}x_{1}^{2} + \frac{{2c_{1} }}{{m_{1} }} + 2\left( { - y_{2} + I_{2} + m_{2} x_{1} } \right)} \right)\left( { - a_{1} x_{1}^{3} + b_{1} x_{1}^{2} - m_{1} x_{1} } \right) + \left( { - \frac{2}{{m_{1} }}\left( {y_{1} + I_{1} + m_{1} x_{2} } \right)} \right)\left( { - y_{1} } \right) + \hfill \\ \, \left( { - 2\left( {y_{1} + I_{1} + m_{1} x_{2} } \right) + \frac{2}{{\varepsilon m_{2} }}\left( {a_{2} + x_{2} } \right)} \right)\left( {x_{2} - b_{2} x_{2}^{3} - m_{2} x_{2} } \right) + \left( {\frac{ - 2}{{m_{2} }}\left( { - y_{2} + I_{2} + m_{2} x_{1} } \right)} \right)\left( { - \frac{{c_{2} }}{\varepsilon }y_{2} } \right) \hfill \\ \end{gathered}$$

From (19) and (22), it is easy to conclude that $\dot{H} = \nabla H^{T} F_{d} \left( x \right)$ which validates the choice of the energy function. Regarding Eq. (16), it is evident that the Hamilton energy of the coupled neurons depends on its state variables (membrane potentials and recovery variables) and its parameters. More interestingly, it is obvious that the energy function defined in Eq. (16) depends on the external currents $I_{1}$ and $I_{2}$ as it was mentioned in some works addressed on the neurons model (Lu et al. 2019; Xin-Lin et al. 2015). Consequently, energy will be sufficient to maintain continuous electrical activities in the considered coupled neurons.

## Result of numerical simulations

### Dynamic behavior of the bidirectional coupled neurons

The well-known fourth-order Runge–Kutta integration algorithm is exploited in this work for various numerical investigations with a fixed step of $5 \times 10^{ - 3}$. Two parameters Lyapunov exponent graphs when increasing respectively decreasing two electrical synapses in both directions enable to quickly explore the various firing activities that can occur into the proposed bidirectional coupled model of neurons.

**Fig. 3:**
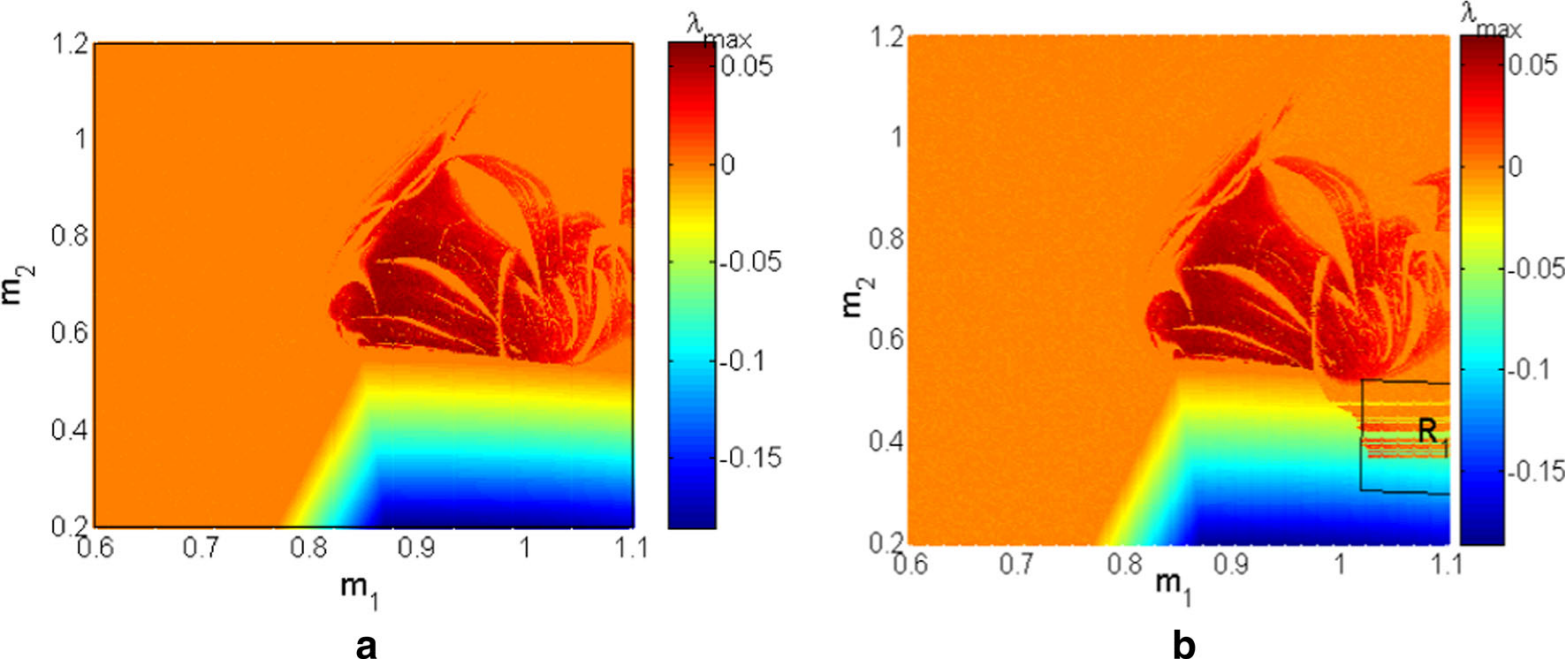
Two parameters Lyapunov exponent of the proposed model when the coupling parameters $m_{1}$ and $m_{2}$ are simultaneously varied in both direction. Initial conditions are $\left( { - 1,2,1,0} \right)$. The left panel **a** is computed by increasing the control parameters and the right panel **b** is computed by decreasing control parameters

**Fig. 4:**
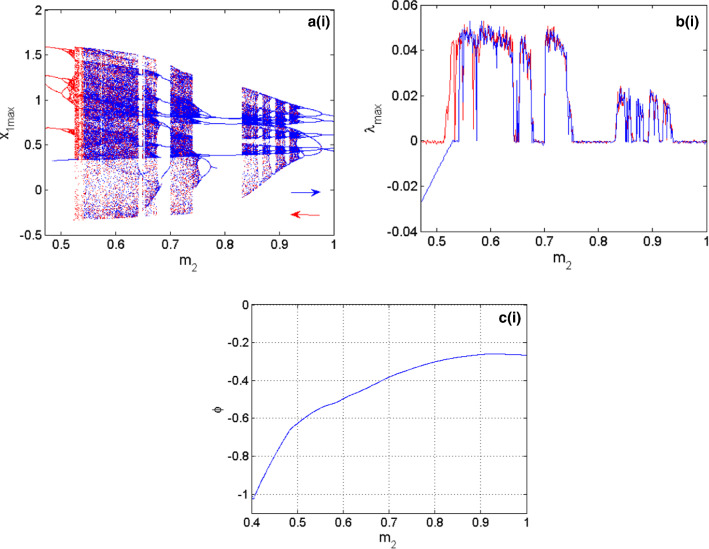
Bifurcations of the coupled neurons with respect to the variation of the synaptic coupling $m_{2}$ in **a** with the corresponding diagram of maximum Lyapunov exponent in **b**. The evolution of the volume contraction rate of the coupled neurons versus $m_{2}$ is depicted in **c**. Initial conditions are $\left( { - 1,2,1,0} \right)$ with $m_{1} = 1$. The diagrams in red are obtained decreasing the synaptic coupling $m_{2}$ while those in blue are obtained when increasing synaptic coupling $m_{2}$

As depicted in Fig. 3a, b the coupled neurons considered in this work can display several types of firing patterns among which resting patterns are characterized by $\lambda_{\max } < 0$, periodic patterns when $\lambda_{\max } = 0$ and chaotic patterns; characterized by $\lambda_{\max } > 0$. In addition, a window of hysteretic neuronal activities can be recorded in the area called $\left( {R_{1} } \right)$ which materializes the region where the right and left diagrams are different. To have a deep and detailed understanding of what happens in our functional coupled neurons, the local maxima of the first neuron are determined. The corresponding bifurcations are computed versus the electrical synapse, which connects the FN neuron with the HR neuron, and the result is presented in Fig. 4a. Figure 4b represents the maximal Lyapunov exponent related to the neuronal activity shown by the bifurcation diagrams. Finally, in Fig. 4c, we have the variation of the volume contraction rate of the model versus the bifurcation parameter $m_{2}$. It is trivial that for any value of $m_{2}$ we have $\phi < 0$ which, justifies the dissipative nature of the proposed coupled neurons model. From Fig. 4a, b two sets of data are clearly identified; the data in blue is obtained when increasing the control parameter and the red data is obtained when decreasing the control parameter. From the blue diagram, the model is able to display three types of neuronal activity including resting patterns characterized by $\lambda_{\max } < 0$, periodic patterns when $\lambda_{\max } = 0$ and chaotic patterns characterized by $\lambda_{\max } > 0$. In contrast, the diagrams in red enable us to identify only two types of firing patterns including periodic patterns for $\lambda_{\max } = 0$ and chaotic patterns for $\lambda_{\max } > 0$. Besides, these various firing activities, a huge window of hysteretic dynamics in the coupled neurons model characterized by the phenomenon of the coexistence of several types of bifurcations is found.

### Hidden coexistence of multiple firing patterns

**Fig. 5:**
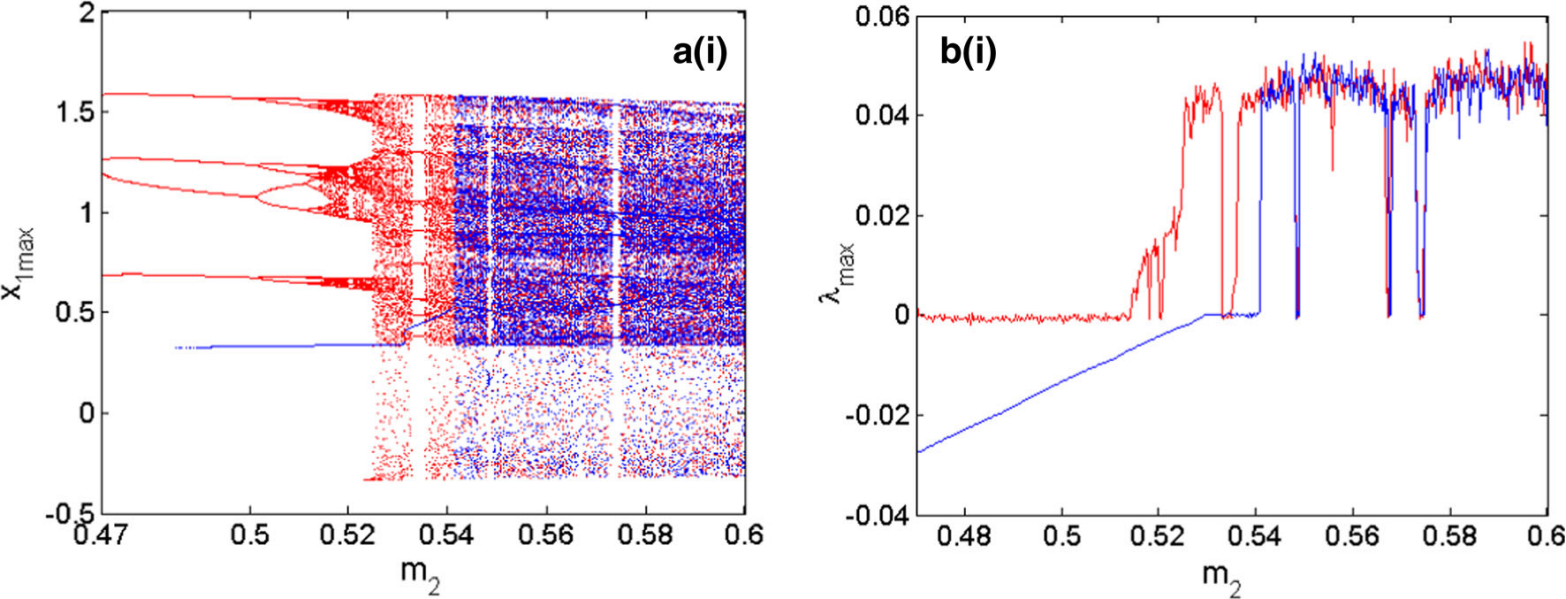
Enlargement of the bifurcation diagrams of Fig. 4a and the equivalent graph of the maximum Lyapunov exponent of Fig. 4b. Initial conditions are $\left( { - 1,2,1,0} \right)$

Multistable dynamics have been investigated in some functional neuron models (Ngouonkadi et al. 2016; Pisarchik et al. 2018; Bao et al. 2019a, b). This phenomenon corresponds to the coexistence of several firing patterns in the state space of a given model of single or coupled neurons. Recall that in practice the initial condition of the neuron can be affected by the environment such as the electric field, the magnetic field (Zhang et al. 2020), the temperature (Xu et al. 2020) as well as the light based on the photosensitivity of the neuron(Liu et al. 2020). The coexisting patterns exhibited by a neuron can be hidden (Bao et al. 2019b) when the neuronal activity comes from either a model with a stable rest point or a model without rest points. Self-excited (Bao et al. 2019a) when the neuronal activity comes from a model with unstable rest points. The phenomenon of coexisting bifurcations exhibited by the coupled neurons model is studied in this work through an enlargement of the window of hysteretic behavior identified on the graphs of Fig. 4. This enlargement is depicted in the graphs of Fig. 5.

The simultaneous study of the bifurcation diagrams of Fig. 5a and the corresponding graphs of the maximum Lyapunov exponents of Fig. 5b allows us to identify many types of activities that coexist. Among others, the coexistence of resting patterns ($\lambda_{\max } < 0$) with periodic patterns ($\lambda_{\max } = 0$), the coexistence of resting patterns ($\lambda_{\max } < 0$) with chaotic patterns ($\lambda_{\max } > 0$) and the coexistence of periodic patterns ($\lambda_{\max } = 0$) with chaotic patterns ($\lambda_{\max } > 0$).

**Fig. 6:**
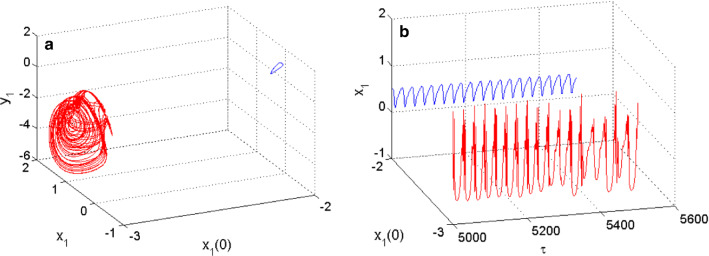
Coexistence of two different types of patterns from the HR neuron including, a periodic spiking pattern (blue) and a chaotic bursting (red) for $m_{2} = 0.54$ using two different initials conditions as depicted on the graph of phase portraits of Fig. 6a and their corresponding time series of Fig. 6b

**Fig. 7:**
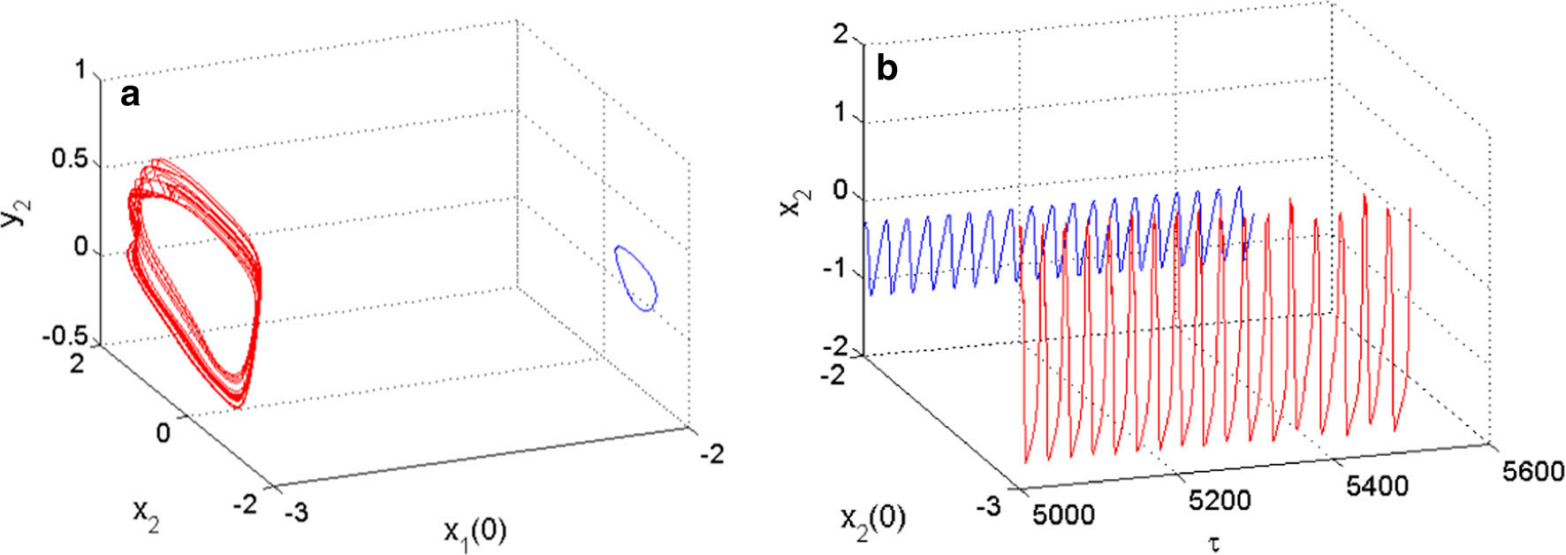
Coexistence of two different types of patterns from the FN neuron including, a periodic spiking (blue) and a chaotic spiking (red) for $m_{2} = 0.54$ using two different initials conditions as depicted on the graph of phase portraits of **a** and their corresponding time series of **b**

**Fig. 8:**
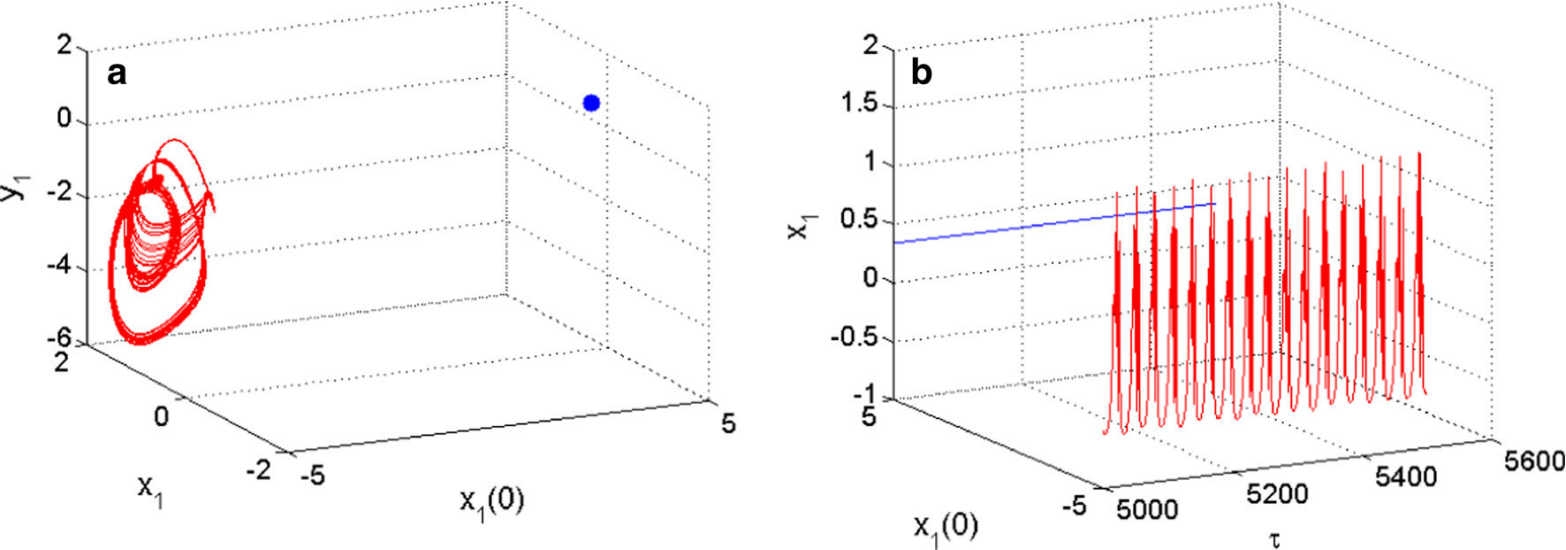
Coexistence of two different types of patterns from the HR neuron including, a resting-state (blue) and a chaotic bursting (red) for $m_{2} = 0.523$ using two different initials conditions as depicted on the graph of phase portraits of **a** and their corresponding time series of **b**

**Fig. 9:**
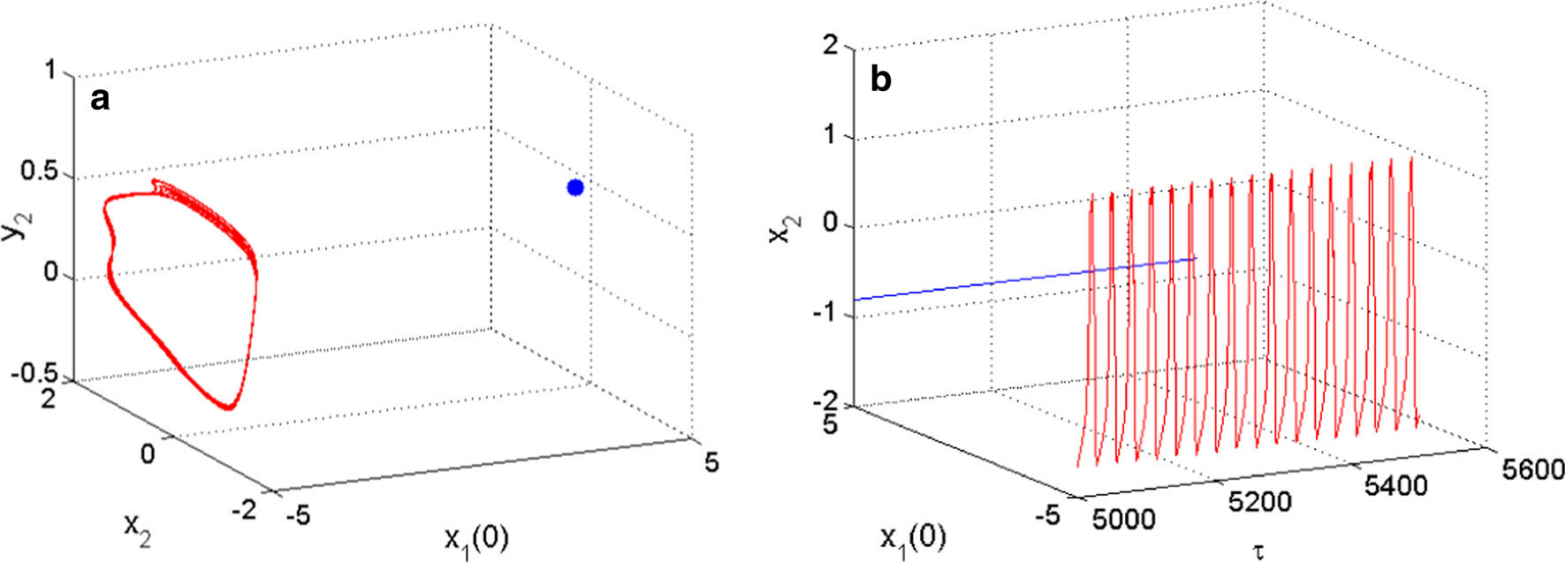
Coexistence of two different types of patterns from the FN neuron including, a resting-state (blue) and a chaotic spiking (red) for $m_{2} = 0.523$ using two different initials conditions as depicted on the graph of phase portraits of **a** and their respective time series of **b**

For example, when $m_{2} = 0.54$, the coupled neurons display the coexistence of periodic spiking and chaotic bursting, as shown in Fig. 6 with the 2D projection of the HR neuron variables. While the coexistence of periodic and chaotic spiking behavior is illustrated in Fig. 7 using the 2D projection of the FN neuron variables as an argument. These coexisting patterns involving periodic and chaotic patterns are supported using both phase portraits in (a) and the time evolution of the membrane potential of each neuron in (b), both for Fig. 6 and Fig. 7. For the set of neurons parameters enabling the neuronal activities of Fig. 6 and Fig. 7, the fixed point of the model is given by $P_{{06}} = \left( {0.{\text{3452,0}}.4042, - 0.{\text{7812, }} - {\text{0}}.0140} \right)$, and the eigenvalues associated with this rest point are given by $\, \lambda_{{1}} { = - 5}{\text{.1889 + 0}}{\text{.0000i;}}$ $\lambda_{{2,3}} { = - 0}{\text{.0855}} \pm {1}{\text{.5975i;}}$ $\lambda_{{4}} { = - 0}{\text{.0774 + 0}}{\text{.0000i}}{.}$ As the coupled neurons model presents eigenvalues with two complex conjugate roots showing negative real parts, and two negative real roots, we can conclude that the model is stable for the considered set of parameters consequently; the coexistence of firing patterns found is hidden. When $m_{2} = 0.523$, the functional model of the coupled neurons under the considerations displays the coexistence of the resting pattern with a chaotic bursting as presented in Fig. 8 using the 2D projection of the HR neuron variable. Furthermore, the coexistence of the resting pattern with a chaotic spiking of Fig. 9 is also obtained for the same set of parameters but using the 2D projection of the FN neuron. These coexisting patterns are further supported using phase portraits as well as the time evolution of the membrane potential of each considered neuron. On the phase space, the resting pattern is characterized by a dot; while on time series is characterized by a straight line.

For these coexisting patterns, the fixed point of the model is given by $$P_{07} = \left( {{0}{\text{.3393,0}}{.4244, - 0}{\text{.7971, - 0}}{.0339}} \right)$$ and the eigenvalues associated given by $$\lambda_{{1}} { = - 5}{\text{.3981 + 0}}{\text{.0000i;}}$$ $$\lambda_{{2,3}} { = - 0}{\text{.0995 }} \pm {1}{\text{.5891i;}}$$$$\lambda_{{4}} { = - 0}{\text{.0767 + 0}}{\text{.0000i}}$$. For this set of parameters, it is clear that the coupled neurons model presents eigenvalues with two complex conjugate roots showing negative real parts, and two negative real roots, and then it is evident that it is stable consequently; display the phenomenon of the coexistence of hidden firing patterns.

### Attraction basins associated with other initials

**Fig. 10:**
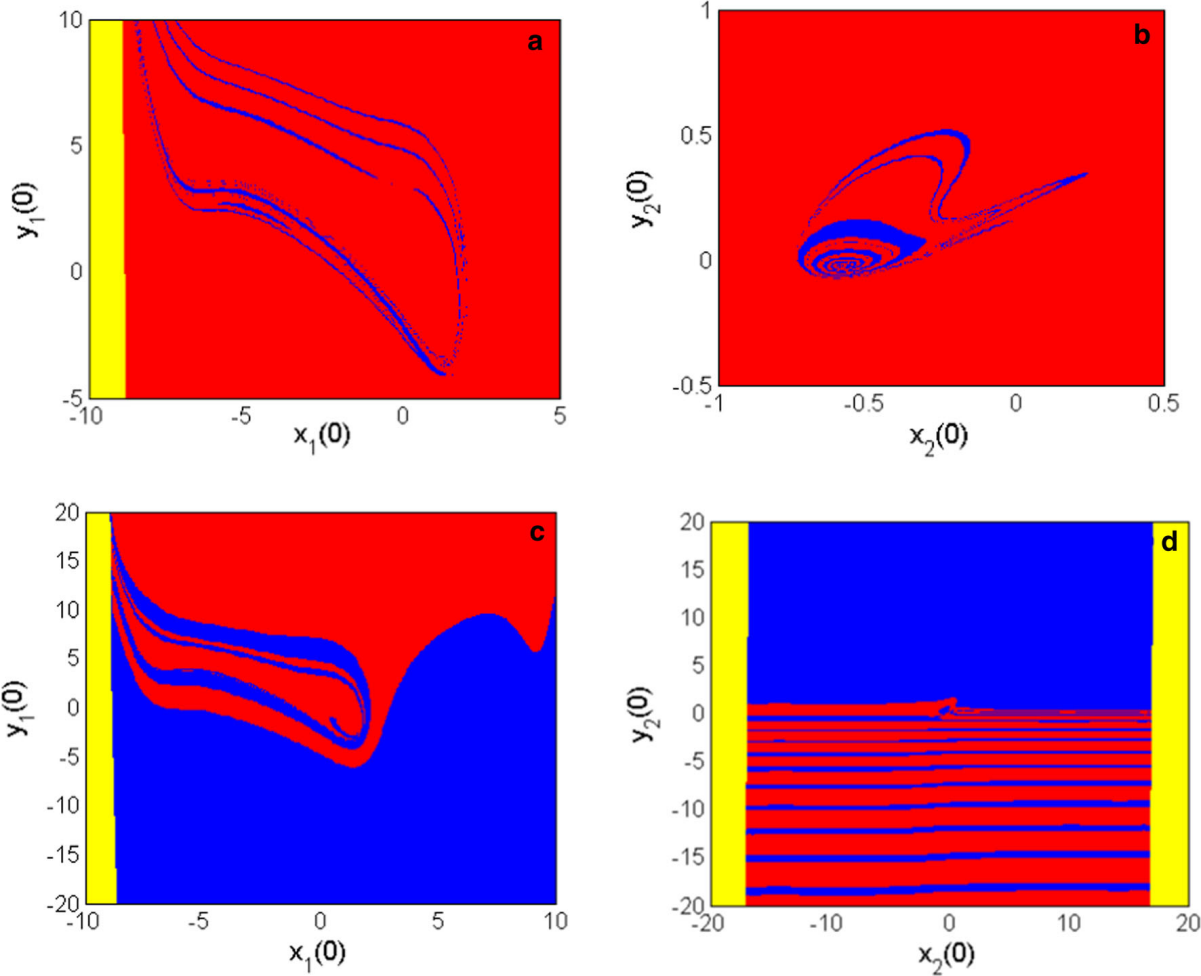
Basin of attractions, given the set of initial conditions associated with each of the firing patterns depicted in Fig. 6–9 for $m_{2} = 0.54$ for **a** and **b** and $m_{2} = 0.523$ for c and d. The red color is associated with the chaotic pattern; the blue color is associated with periodic or resting patterns, while the yellow color corresponds to the unbounded motion of the coupled neurons

The basin of attraction corresponds to the set of initial values leading to each of the various coexisting firing patterns discovered in this work (Kengne et al. 2016a, b; Kengne et al. 2015). When $m_{2} = 0.54$ coexisting behaviors involving periodic and chaotic patterns are captured using both phase portraits and time evolutions as arguments. The basin of attraction of each neuron model (either HR or FN) has been determined as presented in Fig. 10a, b. Figure 10a represents the set of initial conditions; in the $\left( {x_{1} \left( 0 \right),y_{1} \left( 0 \right)} \right)$ plane that enables to obtain; the coexistence of the hidden patterns from the coupled neurons using the HR neuron when the initial conditions of the FN neuron model are all set to zero. While Fig. 10b displays the set of the initial conditions, in the $\left( {x_{2} \left( 0 \right),y_{2} \left( 0 \right)} \right)$ plane that enables the capture of the coexistence of the hidden firing patterns of the coupled neurons from the FN neuron when the initial conditions of the HR neuron are all fixed at zero. When $m_{2} = 0.523$ the functional model of the coupled neurons under consideration displays the coexistence of a resting pattern with a chaotic pattern. The set of the initial conditions; that enables to have each coexisting hidden firing activity are shown in Fig. 10c for the HR neuron and Fig. 10d for the FN neuron. From these figures, the basin of periodic or resting activities and chaotic activities are respectively marked in blue and red while the yellow color stands for the unbounded activity. These attraction basins clearly demonstrate that the distribution of the multistability in each neuron of the coupled model is associated with the initial conditions of the other neuron. In addition, the absence of the riddled structure on each basin of attraction shows that each attraction basin has a specific domain associated with each coexisting pattern thus, the occurrence of extreme events is excluded in the introduced model of coupled neurons (Chaudhuri and Prasad 2014).

## Circuit implementation of the coupled neurons

**Fig. 11:**
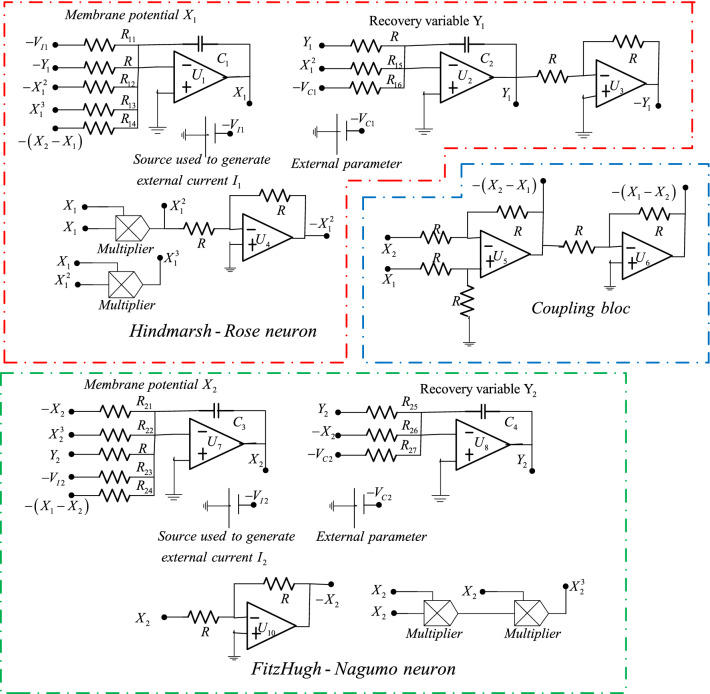
Analog circuit implementation of the bidirectional coupled neurons

In this section, we want to confirm the hidden firing patterns exhibited by the coupled neurons considered in this work. To achieve this goal, an analog circuit based on operational amplifiers TL082, analog multipliers AD633JN, resistors, and capacitors is built as presented in Fig. 11. The power supply of the operational amplifiers is $\pm 15V$ symmetric. The circuit build for the coupled neurons is composed of three main blocks: one block is an emulator of the HR neuron (in the red enclosure), another block serves as an emulator of the FN neuron (in the green enclosure) and the third block plays the role of coupling between the above-mentioned blocks (in the blue enclosure). Using the well-known Kirchhoff’s electrical circuit law, the circuit equations of the coupled neurons are given as:

23 $$\left\{ \begin{gathered} \dot{X}_{1} = Y_{1} - \frac{R}{{R_{{13}} }}X_{1}^{3} + \frac{R}{{R_{{12}} }}X_{1}^{2} + \frac{{V_{{I1}} }}{{R_{{11}} }} + \frac{R}{{R_{{14}} }}\left( {X_{2} - X_{1} } \right) \hfill \\ \dot{Y}_{1} = \frac{R}{{R_{{16}} }}V_{{C1}} - \frac{R}{{R_{{15}} }}X_{1}^{2} - Y_{1} \hfill \\ \dot{X}_{2} = ;\frac{R}{{R_{{21}} }}X_{2} - \frac{R}{{R_{{22}} }}X_{2}^{3} - Y_{2} + \frac{{V_{{I2}} }}{{R_{{23}} }} + \frac{R}{{R_{{24}} }}\left( {X_{1} - X_{2} } \right) \hfill \\ \dot{Y}_{2} = ;\frac{R}{{R_{{27}} }}V_{{C2}} + \frac{R}{{R_{{26}} }}X_{2} - \frac{R}{{R_{{25}} }}Y_{2} \hfill \\ \end{gathered} \right.$$

In the set of the nonlinear differential equation obtained in Eq. (23) $X_{i}$ and $Y_{i}$ represent the state evolution of the membrane potential and the recovery variable respectively. $V_{Ci}$ indicate the outer parameters and $V_{Ii}$ the sources exploited for the generation of external stimulus.

Considering $$RC = 10nF \times 10K\Omega = 100us$$, $$V_{Ci} = \, V_{Ii} = \, V_{0} = \, 1V$$,$$x_{i} = \frac{{X_{i} }}{{V_{0} }}$$, $$y_{i} = \frac{{Y_{i} }}{{V_{0} }}$$.

The comparison between Eq. (23) and Eq. (3) yields to:

$$\frac{R}{{a_{1} }} = R_{13} = {\text{10K}}\Omega$$, $$\frac{R}{{b_{1} }} = R_{12} = {3}{\text{.333K}}\Omega$$, $$\frac{{V_{0} }}{{I_{1} }}R = R_{11} = {\text{25K}}\Omega$$, $$\frac{R}{{m_{1} }} = R_{14} = {\text{10K}}\Omega$$, $$\frac{{V_{c1} }}{{c_{1} }}R = R_{16} = {\text{10K}}\Omega$$, $$\frac{R}{{d_{1} }} = R_{15} = {\text{2K}}\Omega$$, $$R = R_{21} = {\text{10K}}\Omega$$, $$\frac{R}{{b_{2} }} = R_{22} = {\text{30K}}\Omega$$, $$\frac{{V_{0} }}{{I_{2} }}R = R_{21} = \infty$$, $$\frac{R}{{m_{2} }} = R_{24} = tuneable$$ , $$\frac{{\varepsilon V_{c2} }}{{a_{2} }}R = R_{27} = 168.8K\Omega$$, $$\frac{\varepsilon R}{1} = R_{26} {\text{ = 130K}}\Omega$$, $$\frac{\varepsilon R}{{c_{2} }} = R_{25} { = 162}{\text{.5K}}\Omega$$, $$R = 10K\Omega$$, $$C_{i} = 10nF$$.

**Fig. 12:**
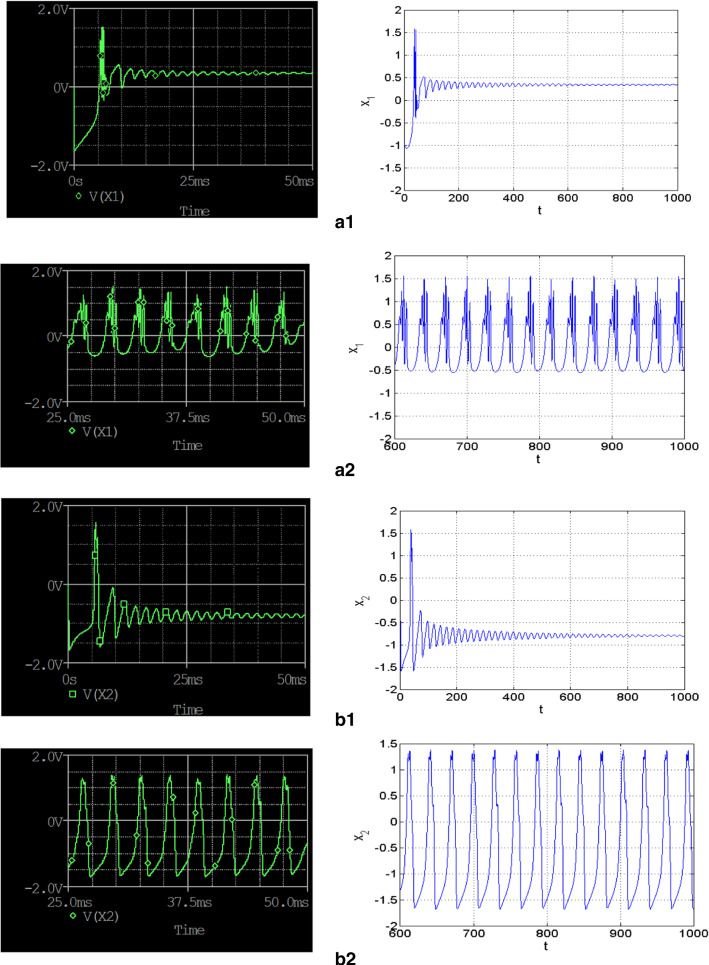
Simulation issues of the analog circuit under PSPICE environment (left-hand side) showing coexisting time evolutions of the membrane potential of the coupled neurons model with their MATLAB equivalents (right-hand side). Those in **a** are associated with the HR neuron while those in **b** are associated with the FN neuron. Initial values of voltages are $\left( {0V,20V,0V,0V} \right)$ for resting behaviors and $\left( {0V, - 20V,0V,0V} \right)$ for the chaotic bursting and spiking behaviors obtained for $R_{24} = 19.1k\Omega$ for $\left( {m_{2} = 0.523} \right)$

When performing PSPICE simulations, the coexisting behaviors found in the previous section have also been reported. Figure 12 shows the coexisting time evolutions of the membrane potentials of the HR neuron in (a) and the one of the FN neuron in (b). From these figures, several kinds of coexisting behaviors are exhibited by coupled neurons. For example, the HR neuron displays the coexistence of the resting pattern with the chaotic bursting behavior while the FN neuron displays the coexistence of the resting pattern with the chaotic spiking behavior. All these coexisting patterns are obtained for the same group of circuit values but using different initial values of the capacitors. Good accordance is observed between numerical analysis (on the right-hand side of Fig. 12) with the PSPICE results (on the left-hand side of Fig. 12).

## Microcontroller based implementation

In this section, the theoretical and numerical dynamics will be reproduced practically using a microcontroller development board (Nestor et al. 2020). A microcontroller is a digital environment system comprising encapsulated in a single case: a microprocessor, memories (Flash, EEPROM, RAM, etc.), and I / O peripherals. This digital computer operates sequentially and can reach operating frequencies of several hundred megahertz. Although it has some drawbacks, the digital computer has several advantages over the analog computer: it is robust, reprogrammable, compact, flexible in setting the parameters and the initial conditions of the oscillator to be implemented with precision, just to name a few. The STM32F407ZE development board was selected as our digital computer due to its important resources, as it is mentioned in Table 2.

**Table 2:** Some important resources of the STM32F407ZE microcontroller for the implementation

Core	ARM Cortex™-M4 32-bit RISC Core
	Floating point unit (FPU)
	Frequency up to 168 MHz
	DSP instructions
	Adaptive real-time accelerator (ART)
Peripheral	Flash memory 512 Kb
	SRAM memory 192 + 4 Kb
	2 × 12-bit D/A converters

**Fig. 13:**
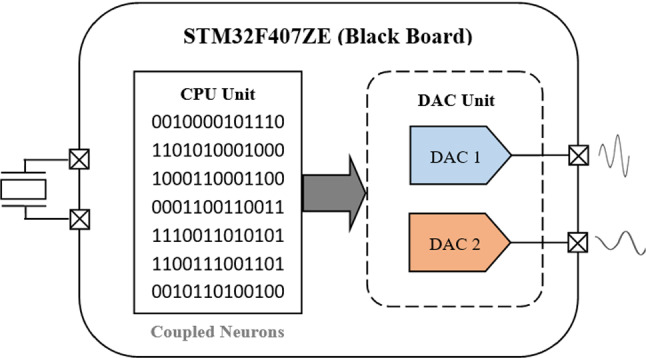
Block diagram of the digital computer with microcontroller (Black Board STM32F407ZE)

The synoptic diagram of our microcontroller computer is given in Fig. 13. This configuration has advantages in terms of cost, size, and gain in energy consumption.

**Fig. 14:**
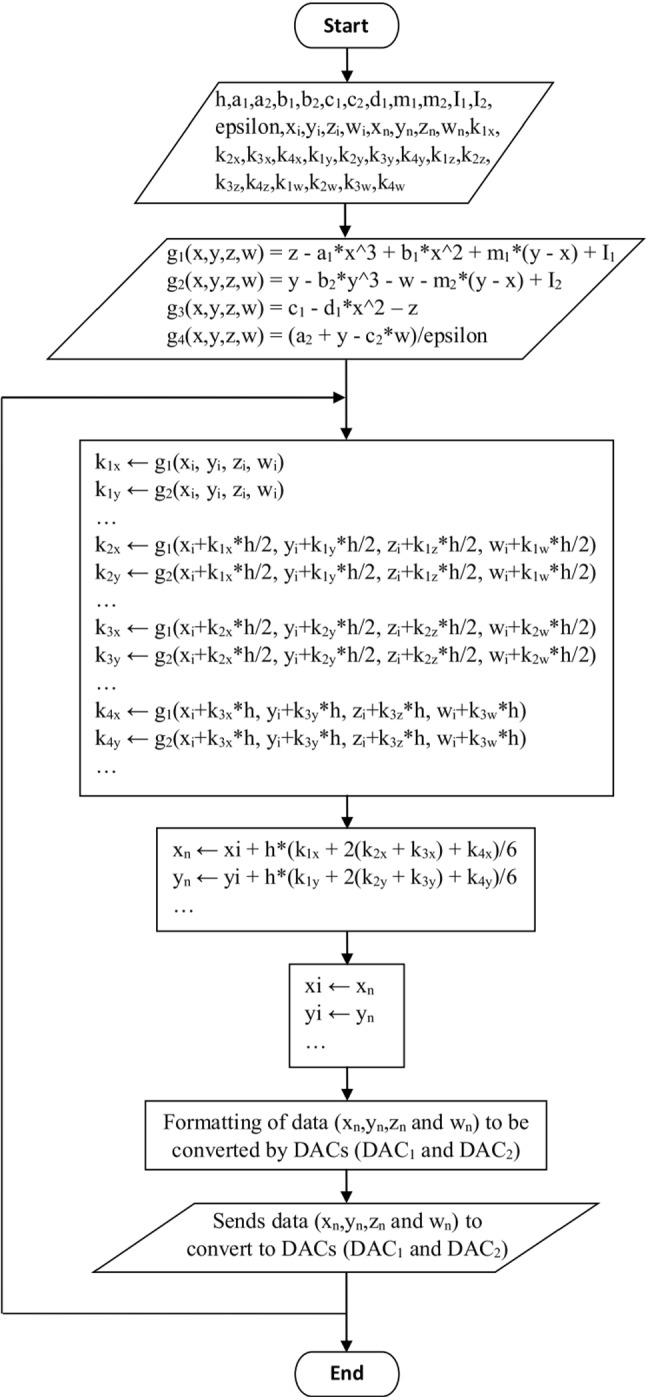
Flowchart of the 4th order Runge–Kutta integration method implemented with the STM32F407ZE

**Fig. 15:**
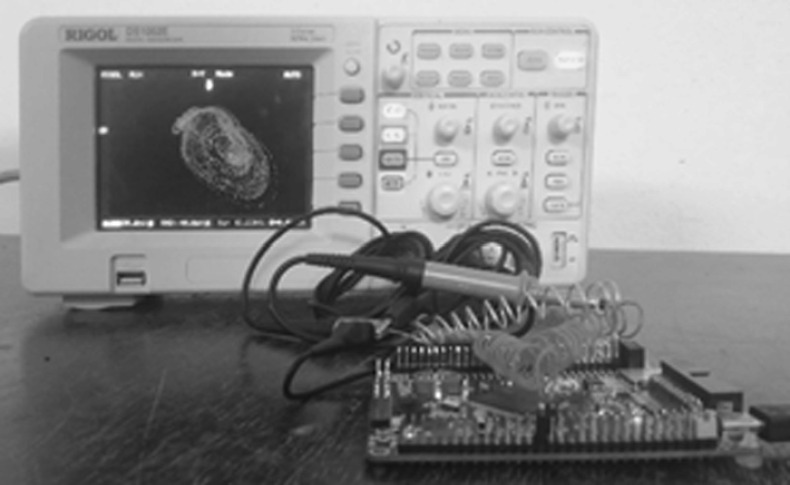
Experimental setup of a digital computer based on a microcontroller and visualization of the signals using a digital oscilloscope

The implementation of the system consists of coding the flowchart of Fig. 14 in an evolved language (here C/C + +) using an integrated development environment (Arduino 1.8.13 in our case). The 4-byte real numbers (float) were used as the data format during experiments. The experimental set-up is presented in Fig. 15 using parameters and initial seed of Fig. 6 and Fig. 7.

**Fig. 16:**
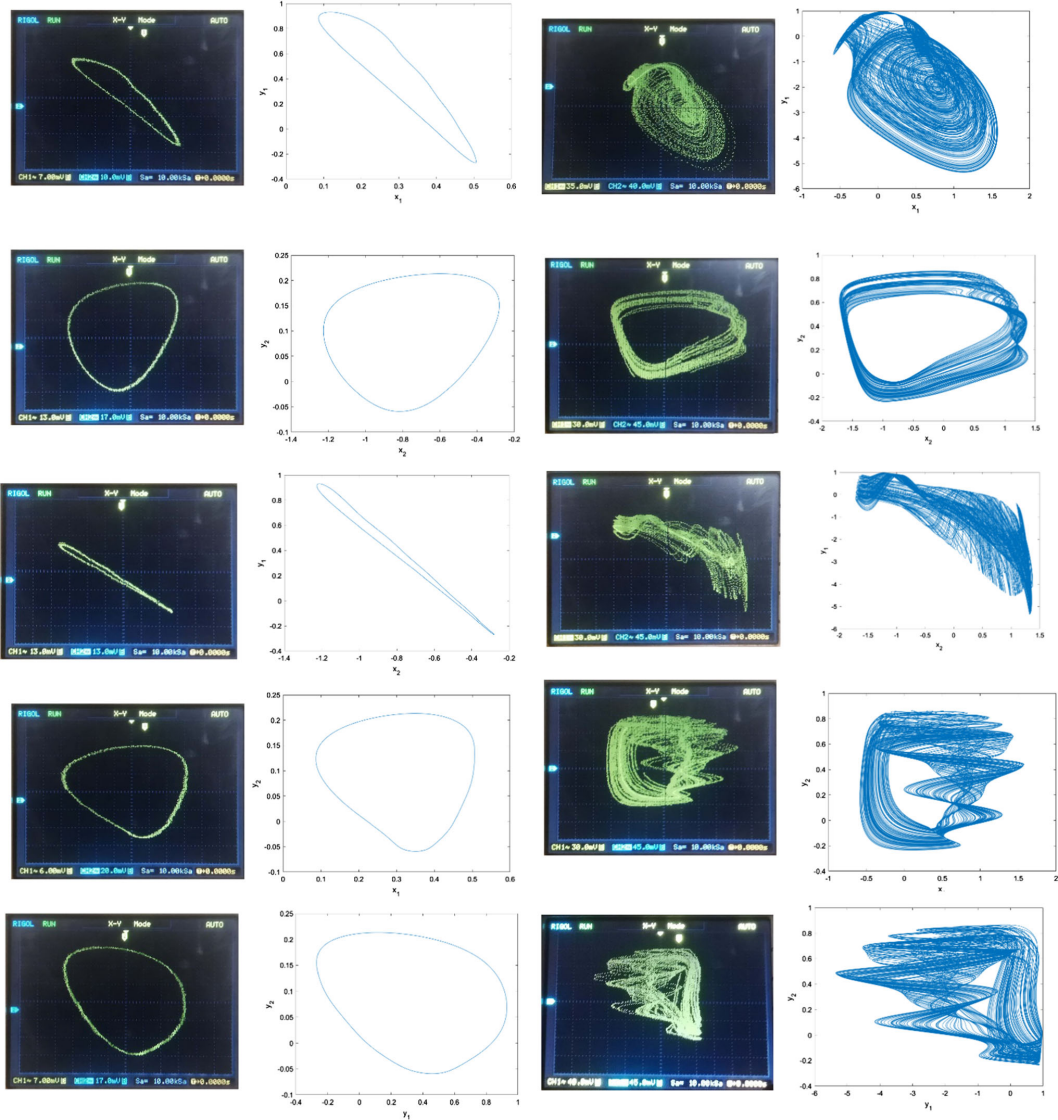
Comparative presentation of the experimental curves (first column and third column) with numerical curves (second column and fourth column). The first two columns are periodic curves while the last two are chaotic curves. Initial conditions and parameters are those of Fig. 6

Following the parameters and initial conditions of Fig. 6, experimental coexisting attractors in several 2D projections, planes are obtained and results are illustrated in figure Fig. 16. High similarity can be observed between the numerical and experimental results.

## Conclusion

In this contribution, a model of bidirectionally coupled neurons consisting of HR and FN neurons was considered and analyzed in depth. The equilibrium point of the coupled neurons was investigated. Their stability has revealed that for some values of the electrical connection, the model under consideration can display either self-excited firing patterns or hidden patterns. In addition, hidden phenomena such as the coexistence of chaotic bursting with periodic spiking, the coexistence of chaotic spiking with period spiking, the coexistence of chaotic bursting with a resting pattern, and the coexistence of chaotic spiking with a resting pattern are also found for some sets of parameters. For all the phenomena reported, the Hamiltonian energy of the model has been determined. It enables us to estimate the amount of energy released during the transition between various electrical activities. A series of PSPICE simulations have been carried out based on the analog circuit of the coupled neurons model to support our numerical results. Finally, a microcontroller implementation based on the STM32F407ZE development board has been used to further support our numerical results of the multistability. Since in real neurons, it is difficult to have the same homogenous dimension in a coupling between several neurons, for future work we are going to explore the complex dynamics of two different families of neurons having different dimensions.

## Data Availability

The data used in this research work are available from the authors by reasonably request.
